# HSP90AA1 interacts with CSFV NS5A protein and regulates CSFV replication *via* the JAK/STAT and NF-κB signaling pathway

**DOI:** 10.3389/fimmu.2022.1031868

**Published:** 2022-11-02

**Authors:** Chenchen Liu, Wei Zhao, Jia Su, Xiaochun Chen, Feifan Zhao, Jindai Fan, Xiaowen Li, Xiaodi Liu, Linke Zou, Mengru Zhang, Zilin Zhang, Liangliang Zhang, Shuangqi Fan, Yuwan Li, Mingqiu Zhao, Jinding Chen, Lin Yi

**Affiliations:** ^1^ College of Veterinary Medicine, South China Agricultural University, Guangzhou, China; ^2^ Key Laboratory of Zoonosis Prevention and Control of Guangdong Province, Guangzhou, China; ^3^ China Institute of Veterinary Drug Control, Beijing, China

**Keywords:** CSFV, HSP90AA1, NS5A, JAK/STAT, NF-κB

## Abstract

Classical swine fever (CSF), caused by the classical swine fever virus (CSFV), is a highly contagious and fatal viral disease, posing a significant threat to the swine industry. Heat shock protein 90 kDa alpha class A member 1 (HSP90AA1) is a very conservative chaperone protein that plays an important role in signal transduction and viral proliferation. However, the role of HSP90AA1 in CSFV infection is unknown. In this study, we found that expression of HSP90AA1 could be promoted in PK-15 and 3D4/2 cells infected by CSFV. Over-expression of HSP90AA1 could inhibit CSFV replication and functional silencing of HSP90AA1 gene promotes CSFV replication. Further exploration revealed that HSP90AA1 interacted with CSFV NS5A protein and reduced the protein levels of NS5A. Since NS5A has an important role in CSFV replication and is closely related to type I IFN and NF-κB response, we further analyzed whether HSP90AA1 affects CSFV replication by regulating type I IFN and NF-κB pathway responses. Our research found HSP90AA1 positively regulated type I IFN response by promoting STAT1 phosphorylation and nuclear translocation processes and promoted the nuclear translocation processes of p-P65. However, CSFV infection antagonizes the activation of HSP90AA1 on JAK/STAT and NF-κB pathway. In conclusion, our study found that HSP90AA1 overexpression significantly inhibited CSFV replication and may inhibit CSFV replication by interacting with NS5A and activating JAK/STAT and NF-κB signaling pathways. These results provide new insights into the mechanism of action of HSP90AA1 in CSFV infection, which abundant the candidate library of anti-CSFV.

## Introduction

Classical swine fever (CSF), caused by the classical swine fever virus (CSFV), is a highly contagious disease in pigs (1), and listed as a notifiable disease by the World Organization for Animal Health (OIE) (2, 3). CSFV, which belongs to the genus *Pestivirus* within the *Flaviviridae* family, is an enveloped virus containing a single-stranded, positive-sense RNA genome of approximately 12.3 kb (4, 5). The genome encodes a poly protein that is processed into 4 structural proteins (C、E^rns^、E1 and E2) and 8 non-structural proteins (N^pro^、p7、NS2、NS3、NS4A、NS4B、NS5A and NS5B) by protease of the virus and host cells (6, 7). These structural and non-structural proteins have been proposed to play diverse roles in proliferation and virulence. Among these proteins, the essential roles of NS5A protein in regulation of viral replication are getting increasing attention with the deepening of research.

CSFV NS5A protein consists of 496 amino acid (aa) residues, with a molecule of mass 55 kDa (8, 9). In spite of the exact function of NS5A is still unknown, many researches on it seem to imply that the NS5A protein is an important tool of CSFV to generate a favorable environment for viral replication. CSFV NS5A protein could interact with a variety of host proteins. For instance, a few studies indicate that NS5A protein interacts with eukaryotic translation initiation factor 3 subunit E (eIF3E), ras-related protein 18 (Rab-18), glucose-regulated protein 78 (GRP78), heat shock protein 70 (Hsp70) and so on to facilitate viral replication (10–13). Recent studies revealed that NS5A induces autophagy to enhance replication of CSFV (14, 15). It has also been shown that CSFV NS5A protein could inhibit the secretion of inflammatory cytokines by suppressing the NF-κB pathway (16). Although the effect of CSFV NS5A on type I IFNs pathway is not well understood, sufficient studies have shown that many flaviviruses NS5 have an antagonism of type I IFN (17). For example, Japanese encephalitis virus (JEV) NS5 could competitively bind to the nuclear transport proteins KPNA3 and KPNA4, which inhibited the nuclear translocation of IRF3 and NF-κB, resulting in the suppression of type I IFN (18). The NS5A protein of hepatitis C virus (HCV) which also belongs to the *Flaviviridae* family, suppresses type I interferon signaling and the phosphorylation of STAT1 (19). Moreover, a study also reveals that binding of the NS5 to Hsp90 could disrupt the interaction of Hsp90 with Janus kinase (JAK), which can help flaviviruses to broadly inhibit JAK/STAT pathway (20).

As is well known, type I IFN (IFN-α/β) is one of the critical lines of defense against viral infections widely (21, 22). IFN-dependent anti-viral response, is initiated by intracellular signaling cascade through the Janus protein kinase (JAK) family members, JAK and Tyk2 (23, 24). The binding of type I IFNs with the receptor (IFNAR) trigger JAK and STATs phosphorylation (25, 26). Subsequently, the phosphorylated STATs dimerize and translocate to the nucleus where they bind to IFN-response elements (ISRE) in ISG promoters to activate transcription of ISGs (24, 26, 27). The antiviral response induced by IFN/JAK/STAT could prevent viral replication directly and quickly (28). ISGs those are amplified effect factors of the IFN signaling cascades have been proved to block various steps of the viral life (26, 29). These ISG-encoded proteins could act directly on the virus to limit viral infection (22, 29). For instance, IFN-α inducible Mx2 could inhibit HBV replication and RNA transcription (30). Studies have shown that ISG15, Mx1 and OAS could inhibit CSFV replication (31, 32).

NF-κB pathway also plays an important role in the control of immunity, inflammation and other processes (33). The binding of viral pathogen-associated molecular patterns (PAMPs) to their receptor host pathogen recognition receptors (PRRs) triggers natural immunity and activates IFN regulatory factor (IRF) family members as well as NF-κB and thus promotes the expression of downstream ISGs (34). The transcription factor NF-κB proteins consist of the Rel family of proteins which include RelA (P65), RelB, c-Rel, p105/p50 (NF-κB1) and p100/p52 (NF-κB2) (35). In most resting cells, the NF-κB protein binds to its inhibitory protein IκB and maintains inactive in the cytoplasm (36). When is activated, the IκB phosphorylates and degrads rapidly, which in turn exposes NF-κB to the nuclear localization sequence (NLS) before translocating to the nucleus to drive the corresponding gene transcription (33). Activation of the NF-κB signaling pathway also play a key role to restrict virus replication (37). Studies have shown that activation of NF-κB is necessary for the generation of ROS to limit HSV-1 replication (38), and may affect HBV viral replication levels by regulating antiviral immunity (39). However, CSFV NS5A inhibits NF-κB nuclear translocation and NF-κB activity (16).

Heat shock protein 90 (HSP90), which family includes HSP90α, HSP90β, glucose-regulated protein 94 (GRP94) and tumor necrosis factor receptor-associated protein 1 (TRAP1) isoforms, is an essential molecular chaperon that is highly conserved in evolution (40, 41). It is involved in diverse biological processes such as virus infection, immune regulation, signal transduction. (42, 43). Many viruses depend on cellular HSP90 to complete their life cycles, especially depend on HSP90α and HSP90β isoforms (44). Studies have revealed that HSP90 is vital for the reverse transcriptase viability of Hepatitis B Virus (HBV), which is essentially required to initiate and maintain HBV reverse transcription (45, 46). In addition to direct binding to viral proteins, HSP90 can also affect the viral infection by regulating the expression of cytokines and antigen presentation (47). It is worth pointing out that HSP90 is required by JAK and IKK to enhance kinase activity, which promotes activation and nuclear translocation of STAT and NF-κB (43, 48).

In previous studies, we found that HSP90AB1 interacts with CSFV NS5A protein (15). However, it is currently unknown whether one of the HSP90 family members, HSP90AA1, also interacts with NS5A and the association of HSP90AA1 with JAK/STAT and NF-κB affects CSFV replication. Therefore, we sought to explore the regulatory effect of HSP90AA1 on JAK/STAT and NF-κB in CSFV infection.

Here, we found that there is a regulatory relationship between CSFV infection and the expression of HSP90AA1. The over-expression of HSP90AA1 inhibits CSFV replication. Further, HSP90AA1 interacts with viral protein NS5A and decreased the protein levels of NS5A. Mechanistically, our results show that over-expression of HSP90AA1 could activate JAK/STAT and NF-κB signaling pathways, which antagonize CSFV infection. Thus, HSP90AA1 is a key host factor limiting CSFV proliferation, it could interact with CSFV NS5A protein and its upregulation could activate host cell antiviral responses.

## Material and methods

### Cells, virus, and plasmids

Porcine alveolar macrophages 3D4/2 were cultured in RPMI 1640 medium (Thermo Fisher, 11875500) with 10% fetal bovine serum (FBS) (Thermo Fisher, 10100147). The swine kidney cell line PK-15 (ATCC, CCL-33) and human embryonic kidney cell line HEK293T (ATCC, CRL-1573) were cultured in Dulbecco’s minimal essential medium (Thermo Fisher, 11965092) with 8% FBS. All cells were cultured at 37°C in a 5% CO_2_ incubator. The CSFV strain (Shimen) used in this study was stored in our laboratory and was propagated in PK-15 cells. pMD18-T-NS5B, p3×Flag-CMV10, pEGFP-C3, pEGFP-NS4A and pEGFP-NS5A were deposited in our laboratory. Lipo3000 (Thermo Fisher, L3000075) reagent was used for transient transfection of plasmids.

### Virus titration by indirect immunofluorescence assay (IFA)

The PK-15 cells were transferred to a 96-well plate, and CSFV virus solution was inoculated when the mono-layer cells reached 70%~80% confluence. The CSFV virus solution was inoculated in 1.5 mL centrifuge tubes with DMEM for 10-fold serial dilution (10-1 to 10-7), and 4 wells were repeated for each dilution.

IFA was used to determine CSFV titers in the culture supernatant. With 48-hour post infection (hpi), cells were washed three times with PBS and fixed with pre-chilled absolute ethanol (200 μL/well) at -20°C for 20 minutes. Following three washes with PBS, the cells were dried at room temperature for about 10 min to completely evaporate the absolute ethanol. The cells were incubated CSFV E2 antibody (JBT, 9011) at 4°C overnight (in dilute E2 protein antibody with PBS at a ratio of 1:200 (40 μL/well). After five washes, the cells were incubated with FITC-labeled or Alexa488-labeled goat anti-mouse IgG antibody (Beyotime, A0428) at 37°C for 1 h. After five washes, Immunofluorescence was observed using a fluorescence microscope (Nikon, Japan). Mock-infected cells were used as controls to establish background staining levels.

### Quantitative real-time RT-PCR (qPCR)

The relative mRNA expression of HSP90AA1, ISGs and IFN-α was tested by RT-PCR using specific primers (**Table 1**). Total RNA from cells was extracted using Total RNA Kit I (OMEGA, R6834) and Viral RNA extraction using Viral RNA Kit (OMEGA, R6874). Subsequently, the cDNA was synthesized by reverse transcription using the HiScript II Q RT SuperMix for qPCR (Vazyme, R223-01). RT-PCR was performed with ChamQ Universal SYBR qPCR Master Mix (Vazyme, Q711-02) according to the manufacturer’s protocol. Relative quantification of mRNA levels was conducted using ΔΔCT method and β-actin as an internal reference gene. Calculation of the gene copy numbers of CSFV was carried out using the absolute quantification method. A standard curve generated from the amplification results of the standard (10-fold serial dilution of the pMD18-T-NS5B plasmid of known concentration) was used to calculate the CSFV gene copy numbers.

**Table 1:** Primers used in this study.

Primers	Sequence (5’∼3’)
NS5B-F	CCTGAGGACCAAACACATGTTG
NS5B-R	GGCACCACACCTTCTACAACGAG
β-actin-F	TCATCTTCTCACGGTTGGCTTTGG
β-actin-R	CCTGACCCTCAAGTACCCCA
HSP90AA1-F	CAGAGGCGGACAAGAACGACAAG
HSP90AA1-R	GATCCTGTTGGCGTGCGTCTG
IFN-α-F	CATCCTGGCTGTGAGGAAATA
IFN-α-R	CAGGTTTCTGGAGGAAGAGAAG
Mx1-F	GAACGAAGAAGACGAATGGAAGG
Mx1-R	GATGCCAGGAAGGTCTATGAGG
OAS2-F	CCAACGGACCCAACCAATAA
OAS2-R	GTCCAGGTGACTCATTCAGAAA
ISG15-F	CTGACCAGTTCTGGCTGACTTTCG
ISG15-R	GCACATAGGCTTGAGGTCATACTCC
Flag-HSP90AA1-F	ACGAATTCAATGCCCGAGGAAACCCA
Flag-HSP90AA1-R	GCTCTAGACTAATCGACTTCCTCCATGCG
siHSP90AA1-F	GGAUCUACAGGAUGAUCAATT
siHSP90AA1-R	UUGAUCAUCCUGUAGAUCCTT

### Western blot analysis

Cell lysates were prepared in radioimmunoprecipitation (RIPA) (Beyotime, P0013) buffer with protease and phosphatase inhibitor cocktail (Beyotime, P1050). The protein concentration was determined with Pierce™ BCA Protein Assay Kit (Thermo Fisher, 23225). The samples were separated by 10% or 12.5% SDS-PAGE that prepared with PAGE Gel Rapid Prep Kit (Jacob enzyme Biotech, PG112) followed by transfer onto polyvinylidene difluoride (PVDF) membranes. After blocking with 5% skim milk at room temperature for 1 h, the membranes were incubated with primary antibodies overnight at 4°C. Primary antibodies used include mouse anti-HSP90AA1 monoclonal antibody (mAb) (Santa Cruz, sc-515081), mouse anti-phospho-STAT1 polyclonal antibody (pAb) (Santa Cruz, sc-136229), mouse anti-tubulin mAb (Beyotime, AT819), mouse anti-Flag mAb (Beyotime, AF5051), rabbit anti-GFP mAb (Beyotime, AF1483), rabbit anti-STAT1 pAb (Beyotime, AF0288), rabbit anti-phospho-JAK1 pAb (Beyotime, AF5857), rabbit anti-P65 mAb (Abmart, AF1234), rabbit anti-JAK1 mAb (Abmart, AT8190), rabbit anti-IκBα mAb (Abmart, T55026S), rabbit anti-phospho-IκBα mAb (Abmart, T57246S), rabbit anti-OAS2 pAb (Sangon Biotech, D121064) and rabbit anti-ISG15 pAb (Sangon Biotech, D225264). After three washes with PBST, the membranes were incubated with horseradish peroxidase (HRP)-conjugated goat anti-mouse IgG (Beyotime, A0216) secondary antibody at 37°C in a thermal shaker for 1 h. Finally, the results were visualized by ECL chemiluminescence kit (Jacob enzyme Biotech, SQ201) and X-ray film exposure (Tanon, China).

### Plasmid construction and synthesis of small interfering RNA

HSP90AA1 PCR amplification primers were designed according to the HSP90AA1 sequence (NM_213973.2) published by NCBI using Primer Premier 5. HSP90AA1 was amplified by PCR from PK-15 cells cDNA and cloned into p3×Flag CMV10. The siRNA targeting HSP90AA1 and a negative control siNC were designed and synthesized by Sangon Biotech. All primers and sequence of siRNA were listed in **Table 1**.

### Immunoprecipitation

The p3×Flag-HSP90AA1 was co-transfected into 293T cells with pEGFP-NS5A or pEGFP-NS4A using Lipofectamine™ 3000, respectively. Controls were represented by cells cotransfected with p3×Flag-HSP90AA1 and pEGFP, p3×Flag-CMV and pEGFP-NS5A. Cells were harvested at 24h after plasmid transfection using Western blot and IP lysis buffer (Beyotime, p0013) containing a protease inhibitor. After centrifugation for 10 min at 4°C, a part of the supernatant was boiled for 10 min with loading buffer (Beyotime, P0015L) as whole cell extracts (Input). The remaining lysate, used for IP experiment, was first incubated with Protein A+G at 4°C with slow rotation for 4h, and then incubated with the corresponding Flag or GFP antibody at 4°C overnight. After incubation, samples were centrifuged at 4°C for 2 min (2000 g/min). The supernatant was discarded and the precipitate was washed five times with pre-cooled PBS. Finally, loading buffer was added to the precipitate and boiled for 10 minutes to perform Western blot experiments with the input samples.

### Confocal microscopy

The HEK-293T cells were cultured in laser confocal dishes to 60% confluence. The p3×Flag-HSP90AA1 was co-transfected into 293T cells with pEGFP-NS5A using Lipofectamine™ 3000, respectively. While setting two controls: 293T cells respectively cotransfected with p3×Flag-HSP90AA1 and pEGFP, p3×Flag-CMV and pEGFP. After culturing for 24 h, the cells were washed twice with PBS and fixed with pre-cooled absolute ethanol at room temperature for 10 minutes. Absolute ethanol was discarded and the cells were washed 3 times with PBS, then permeabilized with 0.1% Triton X-100 for 10 min at room temperature and discarded. Cells were washed 3 times with PBS and incubated with goat anti-mouse Flag antibody (Beyotime, AF5051) overnight at 4°C (antibody was diluted 1:200 in PBS), then washed 3 times with PBS and incubated with Cy3-labeled fluorescent secondary antibody (Beyotime, A0521) at 37°C for 1 Hour. The nucleus was counterstained with DAPI for 10 min. Finally, anti-fluorescence quencher was added dropwise to the cells. Observation of cell fluorescence signals under a laser confocal microscope.

In the experiment of the effect of HSP90AA1 on the phosphorylation and nuclear translocation of STAT1 and P65, p3×Flag-HSP90AA1 was transfected into PK-15 cells and 3D4/2 cells. Primary antibodies include rabbit anti-p-STAT1 pAb (Bioss, bs-3427R), rabbit-anti-p-P65 pAb (Affinity Biosciences, AF2006) and mouse anti-Flag mAb. Fluorescent secondary antibodies include FITC-labeled goat anti-mouse IgG (Beyotime, A0568) and Cy3-labeled goat anti-rabbit IgG (Beyotime, A0516).

## Results

### CSFV infection Up-regulates HSP90AA1 expression

**Figure 1:**
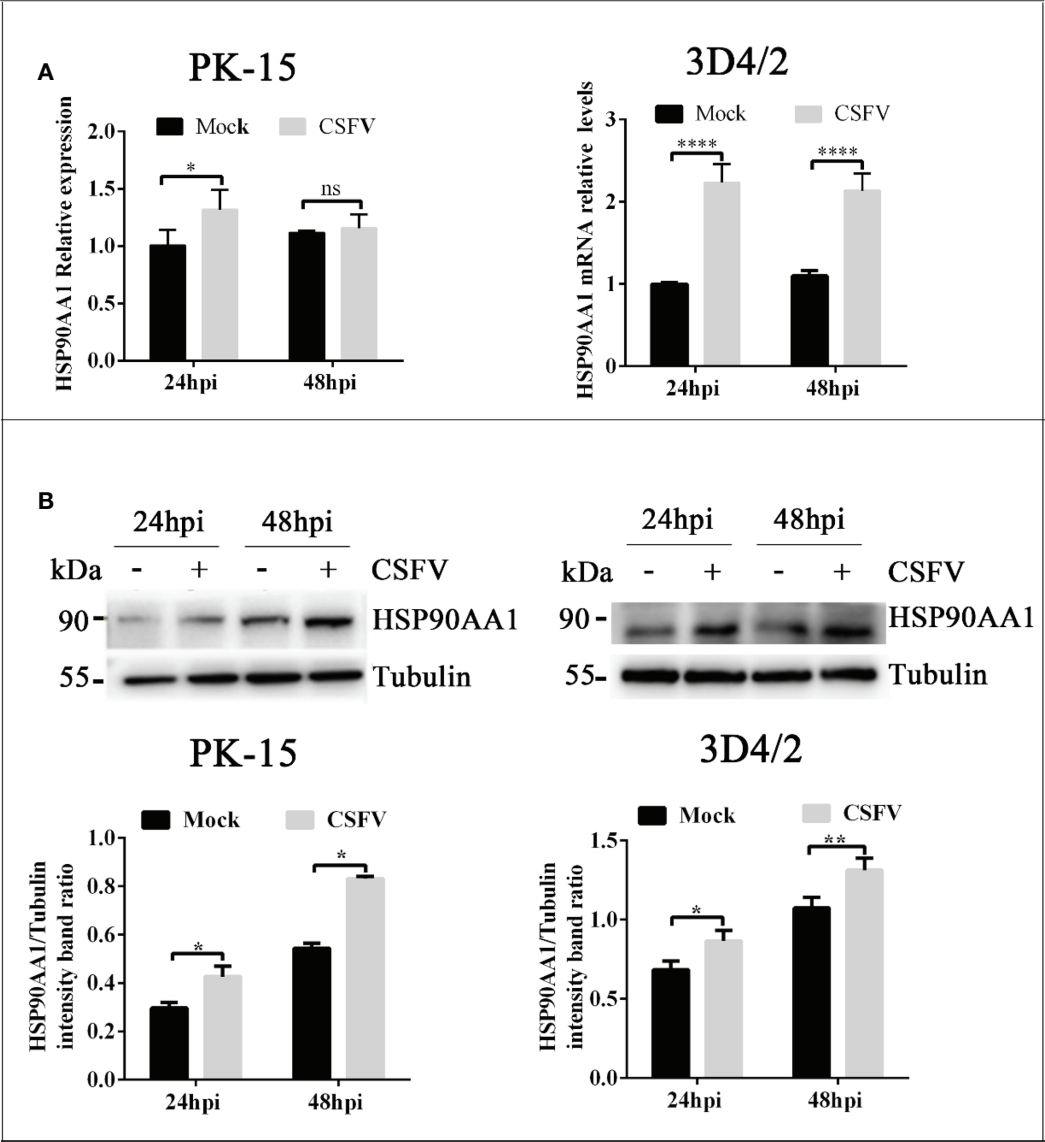
CSFV infection Up-regulates HSP90AA1 expression. **(A)** HSP90AA1 mRNA relative levels in PK-15 and 3D4/2 cells was analyzed by qRT-PCR; **(B)** Western blot showing HSP90AA1 protein expression and the relative protein levels of HSP90AA1 in PK-15 and 3D4/2 cells were estimated by histograms representing density readings of the gel bands with Image J, and the ratios were calculated relative to Tubulin control. (*p < 0.05, **p < 0.01 and ****p < 0.0001 calculated using two-way ANOVA, ns, not significant).

To explore whether CSFV infection affects the expression of HSP90AA1, CSFV-infected (MOI=1) and CSFV-uninfected PK-15 and 3D4/2 cells were harvested at 24 hpi and 48 hpi to detect the mRNA and levels of HSP90AA1. The results showed that there was a significant increase in mRNA and protein levels of HSP90AA1 in PK-15 and 3D4/2 cells compared with control cells (**Figure 1**). The results show that CSFV infection prompted HSP90AA1 expression.

### HSP90AA1 overexpression decreases CSFV replication

**Figure 2:**
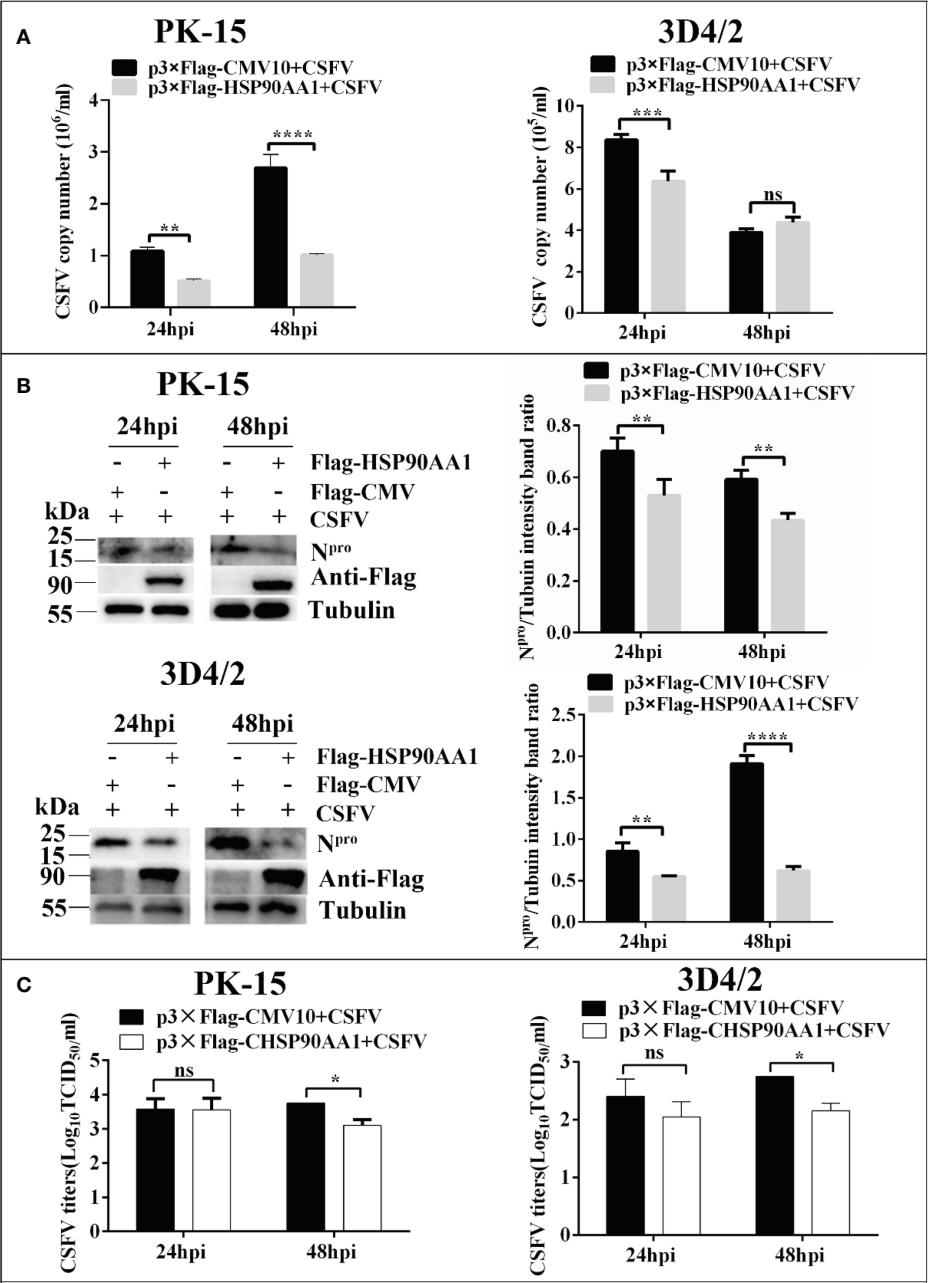
Over-expression of HSP90AA1 inhibits CSFV replication. **(A)** CSFV NS5B copy numbers were determined by RT-PCR at 24 and 48 hpi in HSP90AA1-overexpression PK-15 and 3D4/2 cells. **(B)** Western blot for HSP90AA1 and CSFV N^pro^ expression in HSP90AA1-overexpression PK-15 and 3D4/2 cells, and the relative protein levels of N^pro^ in HSP90AA1-overexpression PK-15 and 3D4/2 cells were estimated by histograms representing density readings of the gel bands with Image J, and the ratios were calculated relative to Tubulin control. **(C)** Infectious progeny viral titers in supernatants from HSP90AA1-overexpressing PK-15 and 3D4/2 cells. Viral titers from the supernatants collected at 24 and 48 hpi were determined and expressed as TCID_50_/ml. (*p < 0.05, **p < 0.01, ***p < 0.001 and ****p < 0.0001 calculated using two-way ANOVA. ns, not significant).

In order to determine the effect of HSP90AA1 on CSFV replication, PK-15 and 3D4/2 cells was transfected with p3×Flag-HSP90AA1 and then infected with CSFV (MOI=1). The replication of CSFV was detected by qRT-PCR, Western blot and IFA, respectively. The results showed that over-expression of HSP90AA1 inhibited CSFV replication in PK-15 and 3D4/2 cells. (**Figure 2**).

### HSP90AA1 inhibition promotes CSFV replication

**Figure 3:**
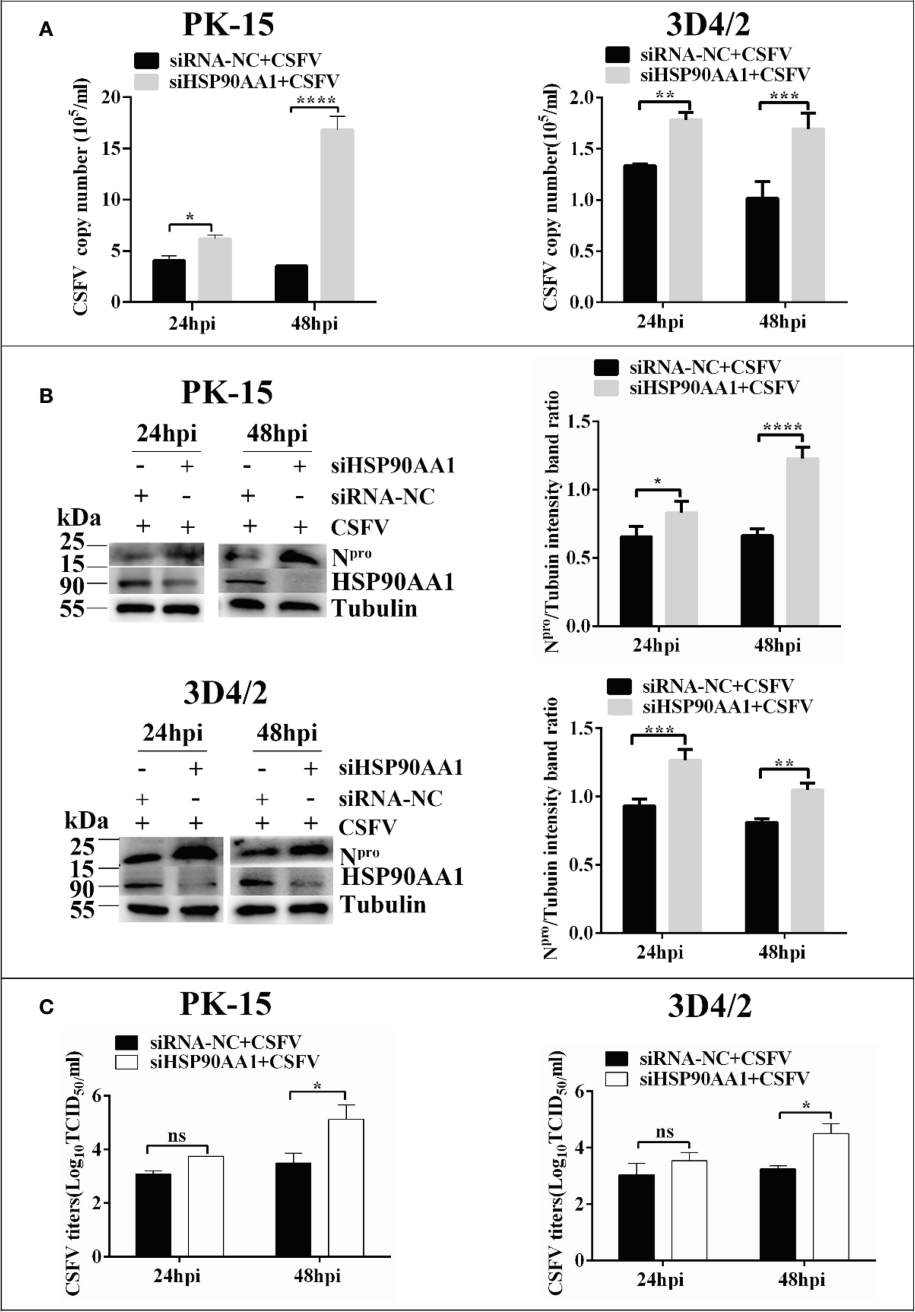
Knockdown of HSP90AA1 promoted CSFV replication. **(A)** RT-PCR determined CSFV NS5B copy numbers at 24 and 48 hpi in HSP90AA1 knockdown PK-15 and 3D4/2 cells. **(B)** Western blot for HSP90AA1 and CSFV N^pro^ expression in HSP90AA1 knock-downed PK-15 and 3D4/2 cells, and the relative levels of N^pro^ in HSP90AA1 knock-downed PK-15 and 3D4/2 cells were estimated by histograms representing density readings of the gel bands with Image J, and the ratios were calculated relative to tubulin control. **(C)** Infectious progeny viral titers in supernatants from HSP90AA1-knockdown PK-15 and 3D4/2 cells. Viral titers from the supernatant collected at 24 and 48 hpi were determined and expressed as TCID_50_/ml. (*p < 0.05, **p < 0.01, ***p < 0.001 and ****p < 0.0001 calculated using two-way ANOVA, ns, not significant).

To investigate whether silencing HSP90AA1 expression affects CSFV replication, small interfering RNA targeting HSP90AA1 (siRNA-HSP90AA1) was transfected into PK-15 and 3D4/2 cells and then the cells were infected with CSFV (MOI=1). Similarly, the replication of CSFV was detected by qRT-PCR, Western blot and IFA, respectively. The results showed that silencing of HSP90AA1 gene function increased CSFV replication in PK-15 and 3D4/2 cells (**Figure 3**).

### HSP90AA1 interacts with CSFV NS5A protein

**Figure 4:**
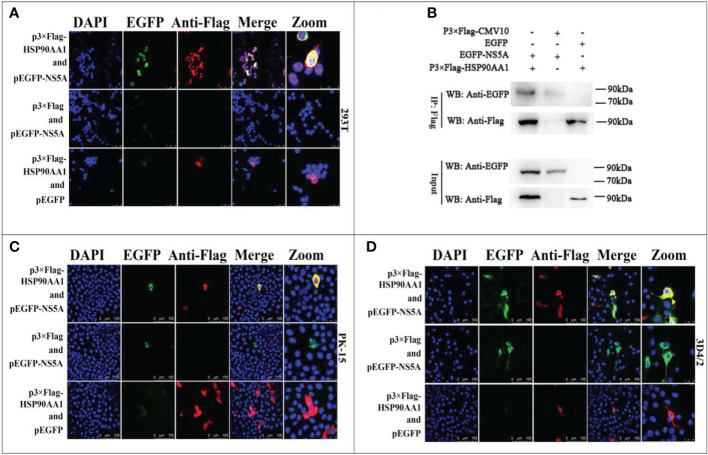
Validation for the interaction of HSP90AA1 with CSFV NS5A protein. **(A)** Identification of the colocalization of HSP90AA1 with NS5A protein in 293T cells. 293T cells co-expressing pEGFP-NS5A and p3×Flag-HSP90AA1 were analyzed by laser-scanning confocal microscopy. 293T Cells were co-transfected with pEGFP and p3×Flag-HSP90AA1 and pEGFP-NS5A with p3×Flag-CMV as negative controls. **(B)** Exogenous Co-IP analysis of NS5A with HSP90AA1 in 293T cells. 293T cells were co-transfected with p3×Flag-HSP90AA1 and EGFP-NS5A. 293T Cells were co-transfected with pEGFP and p3×Flag-HSP90AA1 and pEGFP-NS5A with p3×Flag-CMV as negative controls. **(C, D)** Identification of the colocalization of HSP90AA1 with NS5A protein in PK and 3D4/2 cells. PK-15 and 3D4/2 cells co-expressing pEGFP-NS5A and p3×Flag-HSP90AA1 were analyzed by laser-scanning confocal microscopy. PK-15 and 3D4/2 Cells were co-transfected with pEGFP and p3×Flag-HSP90AA1 and pEGFP-NS5A with p3×Flag-CMV as negative controls.

Our previous works have found that CSFV NS5A protein interacted with HSP90 by liquid chromatography-mass spectrometry (15). Due to that HSP90AA1 was one of the members of the HSP90 family, this study further confirms the interaction of HSP90AA1 with CSFV NS5A protein. The results of confocal laser microscopiclaser showed that CSFV NS5A co-localized with HSP90AA1 in the cytoplasm in 293T, PK-15 and 3D4/2 cells (**Figures 4A, C, D**). And the results of co-immunoprecipitation experiments showed that HSP90AA1 interacted with CSFV NS5A protein in 293T cells (**Figure 4B**).

### HSP90AA1 reduces the protein levels of CSFV NS5A

**Figure 5:**
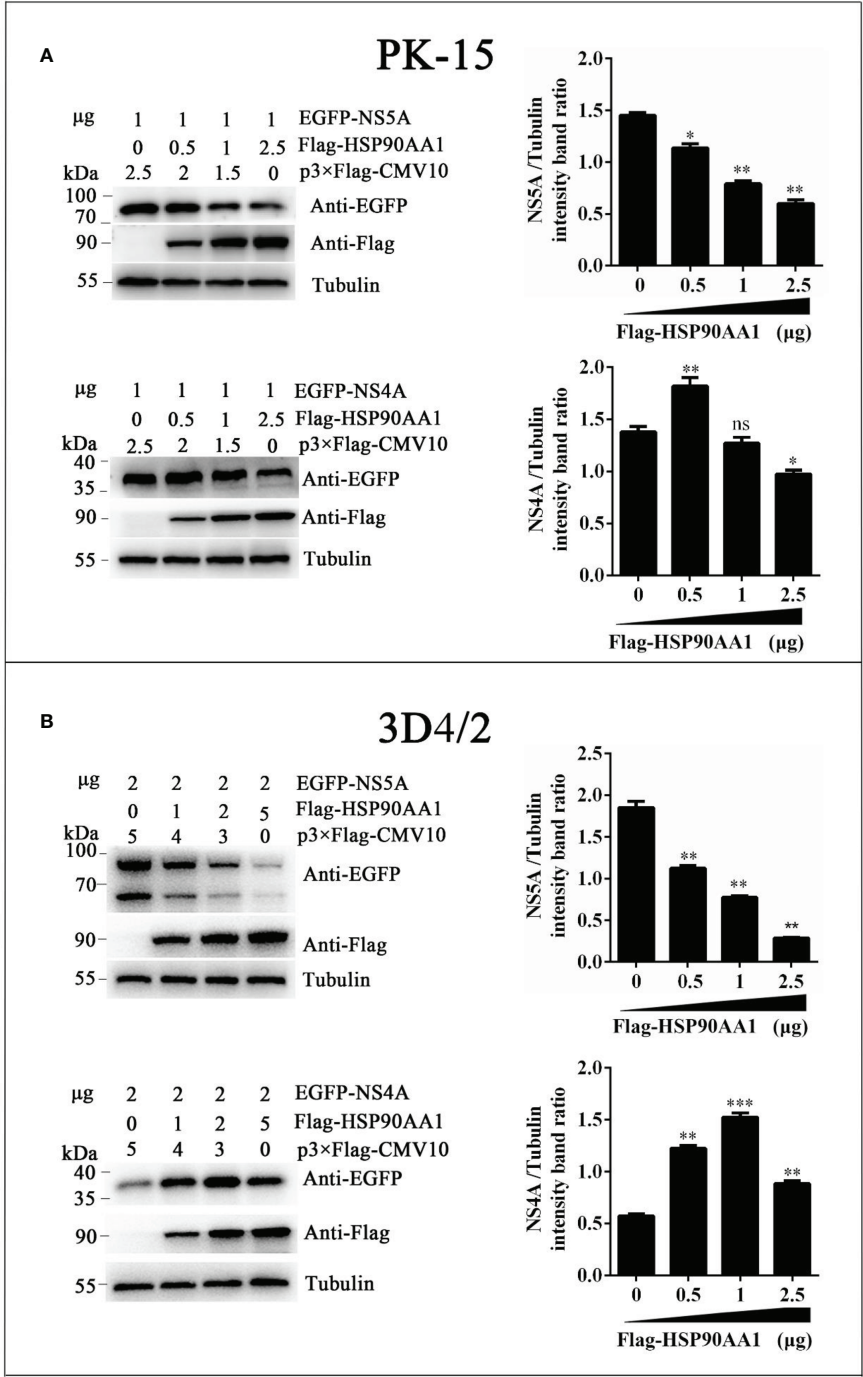
HSP90AA1 reduces the protein levels of CSFV NS5A. **(A, B)** EGFP-NS5A or EGFP-NS4A protein expression in HSP90AA1-expressing PK-15 and 3D4/2 cells. PK-15 and 3D4/2 cells were co-transfected with pEGFP-NS5A or EGFP-NS4A with a different amount of p3×Flag-HSP90AA1. Western blot analyzed EGFP-NS5A or EGFP-NS4A protein expression at 24 hours post co-transfected. Tubulin served as an internal control. The relative levels of EGFP-NS5A or EGFP-NS4A were estimated by histograms representing density reading of the gel bands with Image J, and the ratios were calculated relative to tubulin control. (*p < 0.05, **p < 0.01 and ***p < 0.001 calculated using one-way ANOVA, ns, not significant).

To investigate effects of HSP90AA1 on CSFV NS5A protein levels, we co-transfected pEGFP-NS5A with different amounts of p3×Flag-HSP90AA1 into PK-15 and 3D4/2 cells, and the cells were harvested to assess protein levels of fusion protein EGFP-NS5A by Western blot. Meanwhile, co-transfected NS4A was used as a control. We found that the protein levels of NS5A decreased gradually with the increasing expression of HSP90AA1 in PK-15 and 3D4/2 cells without dose-dependent inhibition of NS4A (**Figure 5**). It suggested that HSP90AA1 was not required to maintain the stability of CSFV NS5A. On the contrary, it can specifically reduce the protein levels of CSFV NS5A.

### HSP90AA1 over-expression activates STAT1 and P65

**Figure 6:**
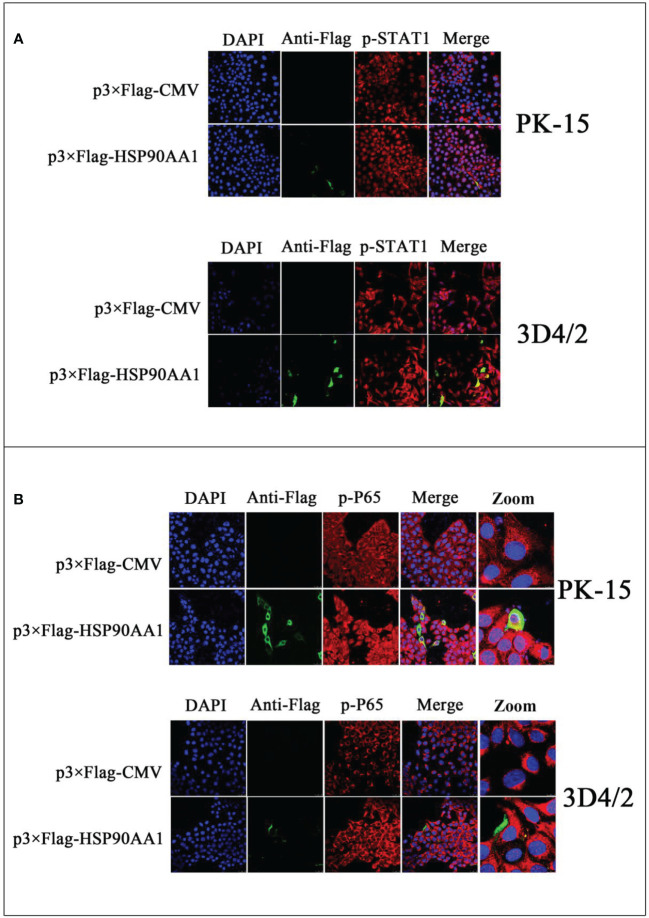
HSP90AA1 over-expression activates STAT1 and P65 in PK-15 and 3D4/2 cells. **(A)** HSP90AA1 over-expression affected the phosphorylation of STAT1 and nuclear translocation of p-STAT1 in PK-15 and 3D4/2 cells were observed by laser confocal microscopy. **(B)** HSP90AA1 over-expression affected the nuclear translocation of p-P65 in PK-15 and 3D4/2 cells were observed by laser confocal microscopy.

To study the effect of HSP90AA1 on JAK/STAT and NF-κB signaling pathways, we detected the phosphorylation and nuclear translocation of STAT1 and P65 in HSP90AA1-overexpressing PK-15 and 3D4/2 cells by laser confocal microscopy. As a result, it was observed that higher levels of p-STAT1 in HSP90AA1-overexpressing PK-15 and 3D4/2 cells than that observed in control cells. And p-STAT1 was mostly distributed in the nucleus of HSP90AA1-overexpressing PK-15 while p-STAT1 in control cells was mainly distributed in the cytoplasm (**Figure 6A**). In addition, the nuclear distribution of p-P65 in HSP90AA1-overexpressing PK-15 and 3D4/2 cells was more than that in control cells (**Figure 6B**). These results indicated that HSP90AA1 over-expression promoted nuclear translocation of p-STAT1 and p-P65.

### HSP90AA1 over-expression does not promote the expressions of ISGs and IFN-α under CSFV infection

**Figure 7:**
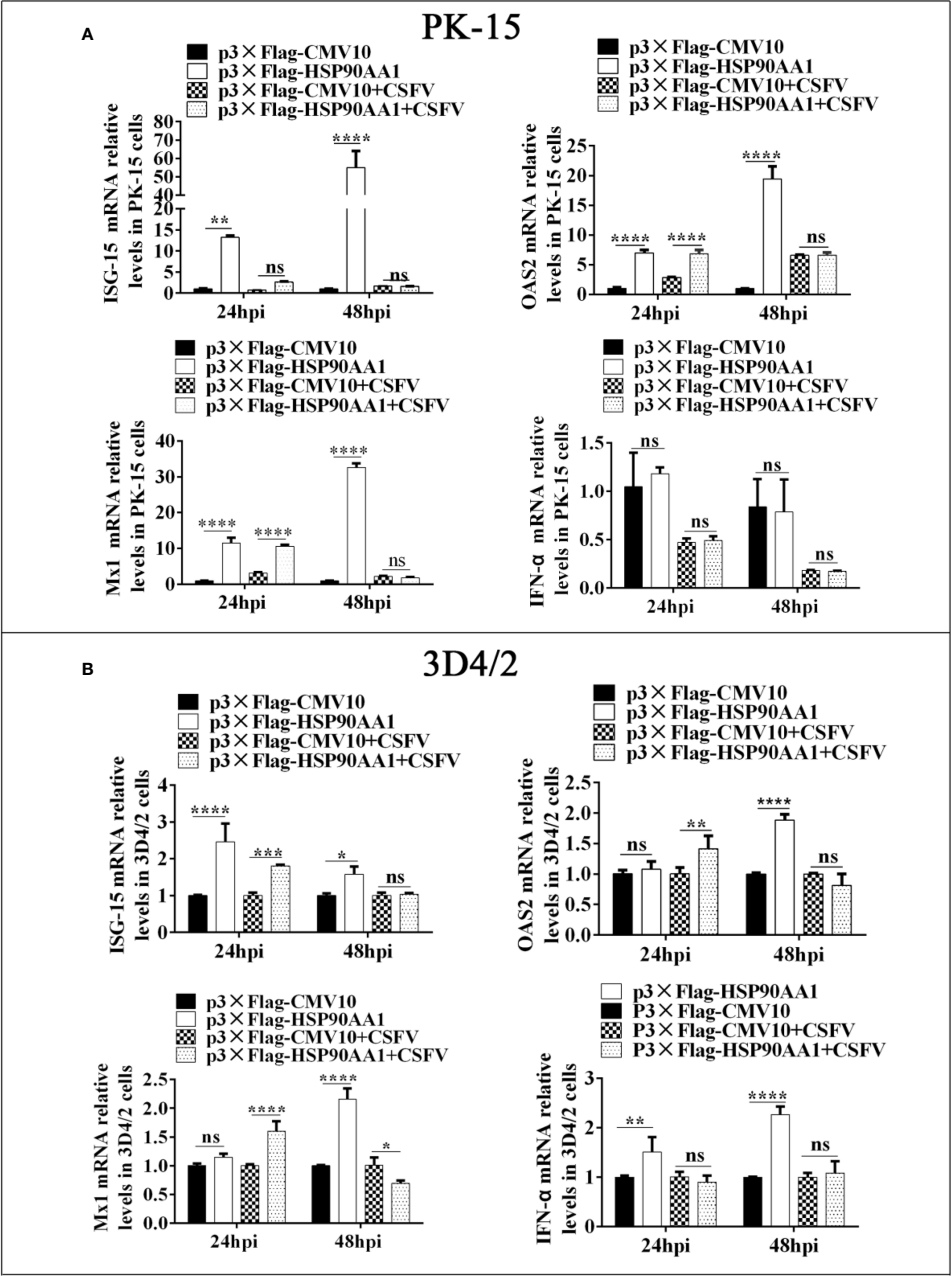
HSP90AA1 over-expression does not promote the expressions of ISGs and IFN-α under CSFV Infection. **(A, B)** Relative mRNA levels of ISGs and IFN-α in PK-15 and 3D4/2 cells were determined by RT-PCR. Cells were transfected with p3×Flag-HSP90AA1 first and then infected with CSFV (MOI=1). Cells were harvested at 24hpi and 48hpi. Total cellular RNA was extracted to determine relative mRNA expression levels of ISGs and IFN-α. (*p < 0.05, **p < 0.01, ***p < 0.001 and ****p < 0.0001 calculated using two-way ANOVA, ns, not significant).

To investigate the effect of HSP90AA1 on the regulation of JAK/STAT and NF-κB pathways under CSFV infection, we first analyzed the expression levels of ISGs and IFN-α in HSP90AA1-overexpressing PK-15 and 3D4/2 cells which were infected with CSFV (MOI=1). The cells were harvested at 24 hpi and 48 hpi to detect the mRNA relative levels of ISGs and IFN-α by RT-PCR. The results showed that over-expression of HSP90AA1 could significantly promote the expressions of ISGs (ISG-15, OAS2 and Mx1) in PK-15 and 3D4/2 cells and significantly promote the expressions of IFN-α in 3D4/2 cells (**Figure 7**). However, HSP90AA1 Over-expression does not promote the expressions of ISGs under CSFV infection (**Figure 7**). This suggests that the facilitation effect of HSP90AA1 on ISGs is diminished or even disappeared after CSFV infection.

### CSFV infection antagonizes the activation of HSP90AA1 on JAK/STAT and NF-κB pathway

**Figure 8:**
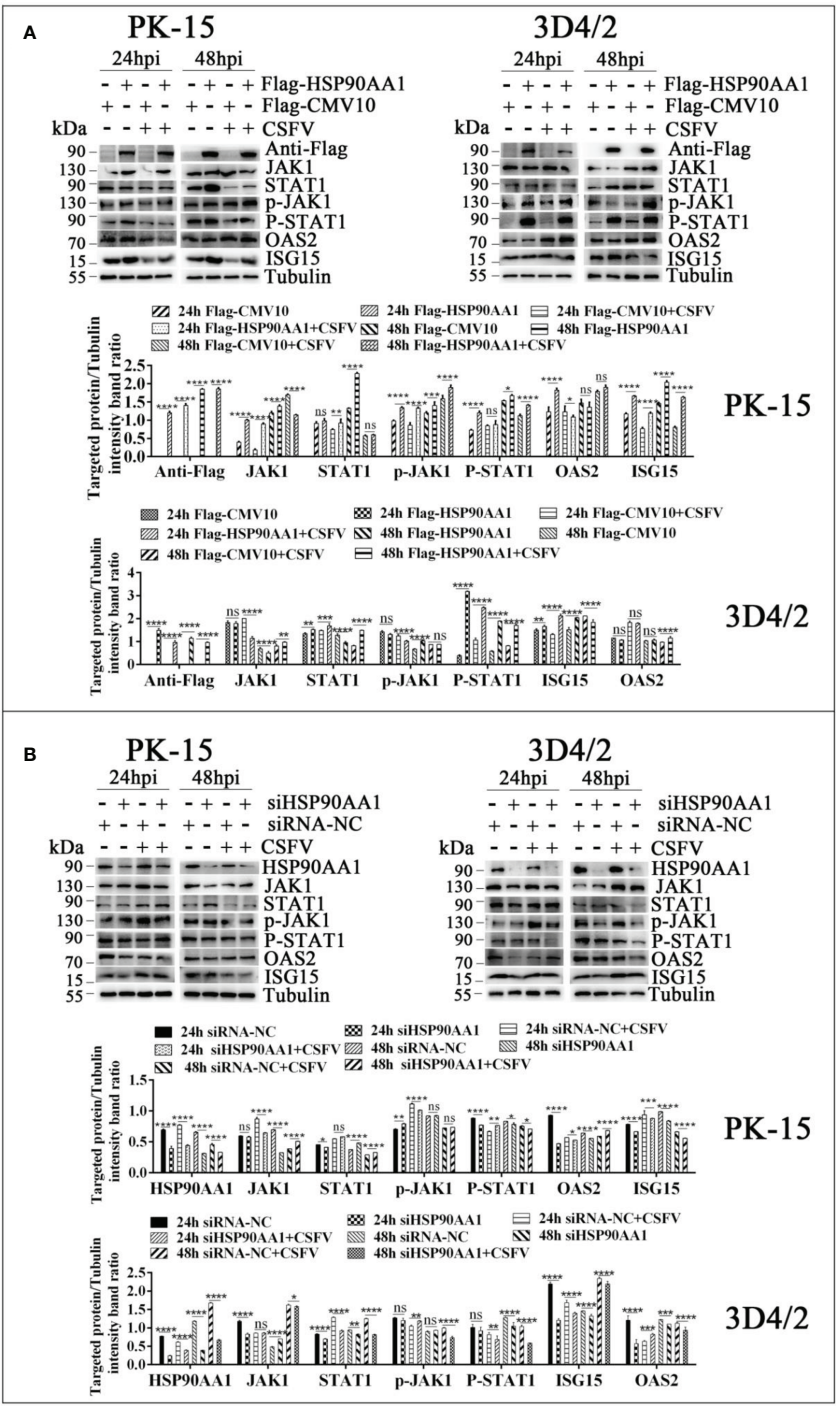
CSFV infection antagonizes the activation of HSP90AA1 on JAK/STAT pathway. **(A, B)** Western blot for JAK1, STAT1, p-JAK1, p-STAT1, OAS2, ISG-15 and HSP90AA1 expression in HSP90AA1-overexpression or knock-downed PK-15 and 3D4/2 cells. Cells were infected with CSFV (MOI=1) after transfection p3×Flag-HSP90AA1 or siHSP90AA1. The cells were not infected with CSFV as a control. Cells were harvested at 24hpi and 48hpi served to Western blot. The relative levels of proteins were estimated by histograms representing density reading of the gel bands with Image J, and the ratios were calculated relative to tubulin control. (*p < 0.05, **p < 0.01, ***p < 0.001 and ****p < 0.0001 calculated using two-way ANOVA, ns, not significant).

**Figure 9:**
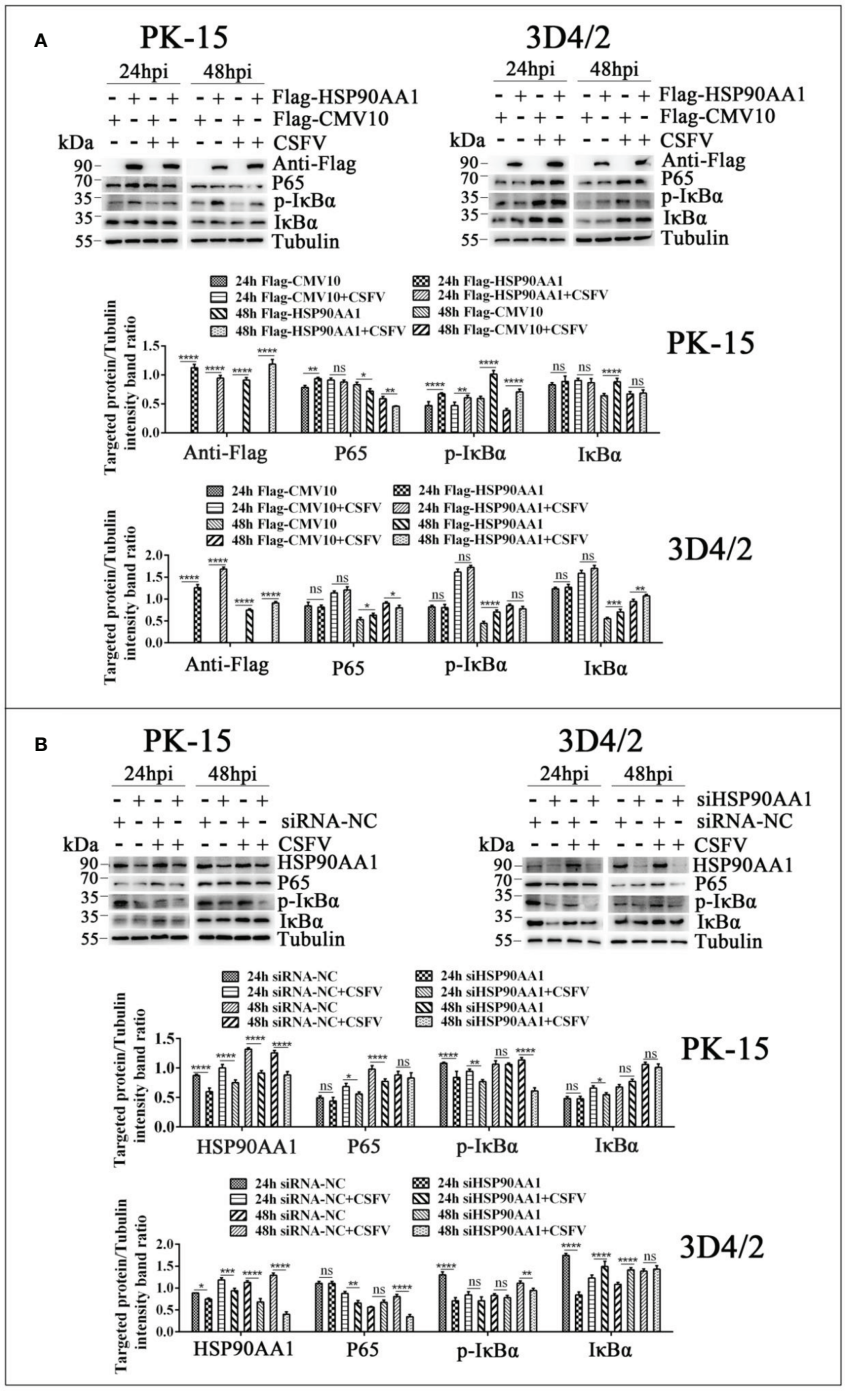
CSFV infection antagonizes the activation of HSP90AA1 on NF-κB pathway. **(A, B)** Western blot for P65, p-IκBα and IκBα in HSP90AA1-overexpression or knock-downed PK-15 and 3D4/2 cells. Cells were infected with CSFV (MOI=1) after transfection p3×Flag-HSP90AA1 or siHSP90AA1, and the cells were not infected with CSFV as a control. Cells were harvested at 24hpi and 48hpi served to Western blot. The relative levels of proteins were estimated by histograms representing density reading of the gel bands with Image J, and the ratios were calculated relative to tubulin control. (*p < 0.05, **p < 0.01, ***p < 0.001 and ****p < 0.0001 calculated using two-way ANOVA, ns, not significant).

To further verify the effects of HSP90AA1 on JAK/STAT and NF-κB pathway under CSFV infection, we detected changes in the levels of proteins related to above two pathways from CSFV infection in HSP90AA1 over-expression or knock-down PK-15 and 3D4/2 cells. The results showed that HSP90AA1 activated JAK/STAT pathway, while CSFV infection attenuates activation of HSP90AA1 on JAK/STAT pathway (**Figure 8**). HSP90AA1 significantly promoted the phosphorylation of IκBα, but had no significant effect on the protein expressions of P65 and IκBα, while CSFV infection inhibited the phosphorylation of HSP90AA1 on IκBα (**Figure 9**). These results showed that CSFV infection antagonizes the activation of HSP90AA1 on JAK/STAT and NF-κB pathway. It is suggested that HSP90AA1 may inhibit CSFV replication *via* activating JAK/STAT and NF-κB pathway.

## Discussion

The production of type I IFNs are triggered by virus infection, which in turn activates the synthesis of interferon-stimulated genes to limit viral proliferation (49). The activation of JAK/STAT and NF-κB signaling pathway plays a key role in establishing the antiviral state (17, 29). However, CSFV suppresses the production of type I IFN, which facilitates its massive replication and persistent infection in tropic cells (15, 50). Moreover, like other members of the *Flaviviridae* family, CSFV NS5A also plays a critical role in regulation of type I IFN response (15, 16). In the current study, we found that HSP90AA1 could inhibit CSFV replication as a host antiviral factor. Further research found that HSP90AA1 may inhibit CSFV replication by inhibiting CSFV NS5A protein expression in a dose-dependent manner and activating JAK/STAT and NF-κB signaling pathway.

The critical role of HSP90 in type I IFN response has been well described (51, 52). HSP90 inhibition can lead to dysregulation of JAK/STAT pathway activation and suppress the activation of NF-κB (53, 54). Our results highlight that HSP90AA1 positively regulates type I IFN response by promoting the stability of JAK, and STAT1 phosphorylation and nuclear translocation processes. It also promotes the activation of NF-kB signaling pathway. However, the promotion of type I IFN and ISGs by HSP90AA1 was inhibited in the CSFV-infected state. We hypothesize that HSP90AA1 is not the main molecule for CSFV to escape innate immunity, there are still other key proteins that help CSFV to escape innate immunity, but HSP90AA1 is important for establishing an intracellular antiviral immune state, which helps to limit viral infection.

Like other members of the *Flaviviridae* family, CSFV infection significantly inhibits the activation of the JAK/STAT signaling pathway (50, 55). Although the mechanism of the inhibition is not fully elucidated, many studies suggest that flavivirus nonstructural protein NS5 plays an important role in this process (56–58). Justin A. et al. proposed that the binding of the flavivirus nonstructural protein NS5 to HSP90 resulting in an imbalance in JAK/STAT pathway signal transduction (20). Our results show that HSP90AA1 is not necessary to maintain NS5A stability, but rather dose-dependently inhibits the protein levels of NS5A. It suggests that HSP90AA1 can act as a key host restriction factor to limit CSFV replication by inhibiting the CSFV NS5A protein.

It seems to be evident that HSP90 is able to enhance the homeostasis of signal transduction proteins in stressful stress states (42, 59). However, there are also distinct effects of HSP90 in different viral infections. It has been shown that HSP90AA1 can promote rabies virus (RABV) proliferation by binding to P protein (60). Enterovirus 71 (EV71) in human rhabdomyosarcoma enters cells by binding to HSP90β on the surface of cells and the cytoplasmic HSP90β can prevent viral proteins from being degraded by proteasome (61). However, our results revealed that CSFV infection induces upregulation of HSP90AA1 expression and that HSP90AA1 overexpression activates innate immunity to in turn inhibit CSFV replication. We hypothesize that inhibition of type I interferon response by CSFV infection may lead to the accumulation of HSP90AA1, which has a regulatory effect on innate immunity. Although upregulation of HSP90AA1 did not alter the inhibition of the type I interferon response by CSFV, it may act as a negative feedback signal to promote cell survival and protect cellular proteins from the risk of damage. Moreover, our study found that HSP90 is not essential for protein stabilization of CSFV NS5A of the *Flaviviridae* family, but restricts CSFV infection by activating the type I IFN signaling pathway. It shares similarities with the involvement of HSP90 in the regulation of cellular and inflammatory factors during flavivirus infection (20). It suggests that the role of HSP90 in flavivirus infection may be specific. It remains relevant to explore the important mechanisms of HSP90 action during flavivirus infection.

The important role of molecular chaperone proteins like HSP70 and HSP90 in viral infections has been studied extensively and heat shock proteins are very conserved in evolution (62, 63). Therefore, some researchers have also suggested that inhibitors of the above two chaperone proteins can be used as broad-spectrum antiviral drugs (64, 65). However, the mechanisms of regulation of viral homeostasis and regulation of intracellular protein homeostasis by molecular chaperones are complex and even opposite for different viral infection processes. So, it is very necessary to investigate the mechanism of interaction between heat shock proteins and flavivirus proteins and the effects of the interaction on immune and inflammatory. It can provide new ideas to elucidate the molecular mechanism of viral infection and help to screening of broad-spectrum anti-flavivirus drugs.

## Data availability statement

The datasets presented in this study can be found in online repositories. The names of the repository/repositories and accession number(s) can be found in the article/supplementary material.

## Author contributions

CL, SF, JF, and JC conceived and designed the study. CL, MeZ, XLiu, FZ, LZo, ZZ, WZ, JS, and LZh performed the experiments. CL, SF, XLi, XC, YL, and JF analyzed the data. CL, SF, MiZ, LY, and JC wrote the manuscript. All authors read and agreed upon the final manuscript.

## Funding

This work was supported by the Program of National Natural Science Foundation of China (No.321728243、No.32102643), the Science and Technology Program of Guangzhou, China, (No. 202206010161), the Key Research Projects of Universities in Guangdong Province (No.2019KZDXM026), Quality and Efficiency Improvement Project of South China Agricultural University(No.C18).

## Conflict of interest

The authors declare that the research was conducted in the absence of any commercial or financial relationships that could be construed as a potential conflict of interest.

## Publisher’s note

All claims expressed in this article are solely those of the authors and do not necessarily represent those of their affiliated organizations, or those of the publisher, the editors and the reviewers. Any product that may be evaluated in this article, or claim that may be made by its manufacturer, is not guaranteed or endorsed by the publisher.
